# In the March 2016 issue

**DOI:** 10.1590/S1980-57642016DN10100001

**Published:** 2016

**Authors:** Ricardo Nitrini

In this issue we are publishing one review, eight original papers, one history note and one case report. 

Rimkus et al. reviewed the association between cognitive impairment in multiple sclerosis (MS) and magnetic resonance imaging (MRI), demonstrating that a more comprehensive MRI approach is needed to better understand the relationship between both white and gray matter disease and cognitive decline in MS. 

Pinto et al. used the Phototest, a brief cognitive test, to investigate the presence of cognitive impairment in MS patients. This study revealed the high accuracy of the Phototest, which represents an option for neurocognitive screening in MS.

Beckert et al. investigated the influence of depression on the performance of low-educated elderly in Addenbrooke's cognitive examination-revised (ACE-R) Test. In this prospective case-control study, depressive symptoms had no influence on ACE-R test scores.

Steibel et al. evaluated the influence of aging and education on the performance of Brazilian elderly in the Rivermead Behavioral Memory Test (RBMT), an ecological test simulating simple everyday activities. Both age and level of education were found to have a significant impact on test performance. 

Beber and Chaves analyzed whether the performance of Brazilian elderly on the verb fluency test was influenced by previous administration of other verbal fluency tests. The authors observed no effect of previous test administration but noted a strong influence of educational level on verb fluency test scores. 

Jacinto et al. evaluated the knowledge and attitudes of medical residents in relation to dementia in a university-hospital using a questionnaire. Medical residents held poor knowledge about epidemiology of dementia, but adequate attitudes concerning disease management and diagnosis. 

Souza et al. evaluated three screening tools for the detection of HIV-associated neurocognitive disorder (HAND): the International HIV-Dementia Scale (IHDS), Mini-Mental State Examination (MMSE) and a self-perception questionnaire. There was low accordance among these screening tools in this sample. 

Costa-Silva et al. investigated the cognitive performance of adolescents with migraine using standardized neuropsychological tests. Cognitive dysfunctions were common in adolescents with migraine and these dysfunctions proved similar to those described in adults with migraine. 

De Paula et al. investigated the utility of a "simplified" version of the Taylor complex figure test (TCFT) for the assessment of older adults with low educational background and provided preliminary evidence of its psychometric properties.

Engelhardt reviewed the first descriptions of the hippocampus in this historical note. Arantius, a pupil of Vesalius, was the first to describe the structure, for which he also provided the name hippocampus. Duvernoy was the first to depict this structure in a publication. 

Soares-Neto et al. reported a case of limbic encephalitis in a patient with lymphoma who manifested Delusional Misidentification Syndrome as one of the most significant behavioral changes. 

Ricardo Nitrini Editor-in-Chief

